# Inter-relationship between subtropical Pacific sea surface temperature, Arctic sea ice concentration, and North Atlantic Oscillation in recent summers

**DOI:** 10.1038/s41598-019-39896-7

**Published:** 2019-03-05

**Authors:** Young-Kwon Lim, Richard I. Cullather, Sophie M. J. Nowicki, Kyu-Myong Kim

**Affiliations:** 1 Global Modeling and Assimilation Office, NASA Goddard Space Flight Center, Greenbelt, MD 20771 USA; 2 Cryospheric Sciences Laboratory, NASA Goddard Space Flight Center, Greenbelt, MD 20771 USA; 3 Climate and Radiation Laboratory, NASA Goddard Space Flight Center, Greenbelt, MD 20771 USA; 4 Goddard Earth Sciences Technology and Research/I. M. Systems Group, Greenbelt, MD 20771 USA; 5 Earth System Science Interdisciplinary Center, University of Maryland, College Park, MD 20742 USA

## Abstract

The inter-relationship between subtropical western–central Pacific sea surface temperatures (STWCPSST), sea ice concentrations in the Beaufort Sea (SICBS), and the North Atlantic Oscillation (NAO) in summer are investigated over the period 1980–2016. It is shown that the Arctic response to the remote impact of the Pacific SST is more dominant in recent summers, leading to a frequent occurrence of the negative phase of the NAO following the STWCPSST increase. Lag–correlations of STWCPSST positive (negative) anomalies in spring with the negative (positive) NAO and SICBS loss (recovery) in summer have increased over the last two decades, reaching *r* = 0.4–0.5 with significance at the 5 percent level. Both observations and the atmospheric general circulation model experiments suggest that the positive STWCPSST anomaly and subsequent planetary-scale wave propagation act to increase the Arctic upper-level geopotential heights and temperatures in the following season. This response extends to Greenland, providing favorable conditions for developing the negative phase of the NAO. Connected with this atmospheric response, SIC and surface albedo decrease with an increase in the surface net shortwave flux over the Beaufort Sea. Examination of the surface energy balance (radiative and turbulent fluxes) reveals that surplus energy that can heat the surface increases over the Arctic, enhancing the SIC reduction.

## Introduction

**Figure 1:**
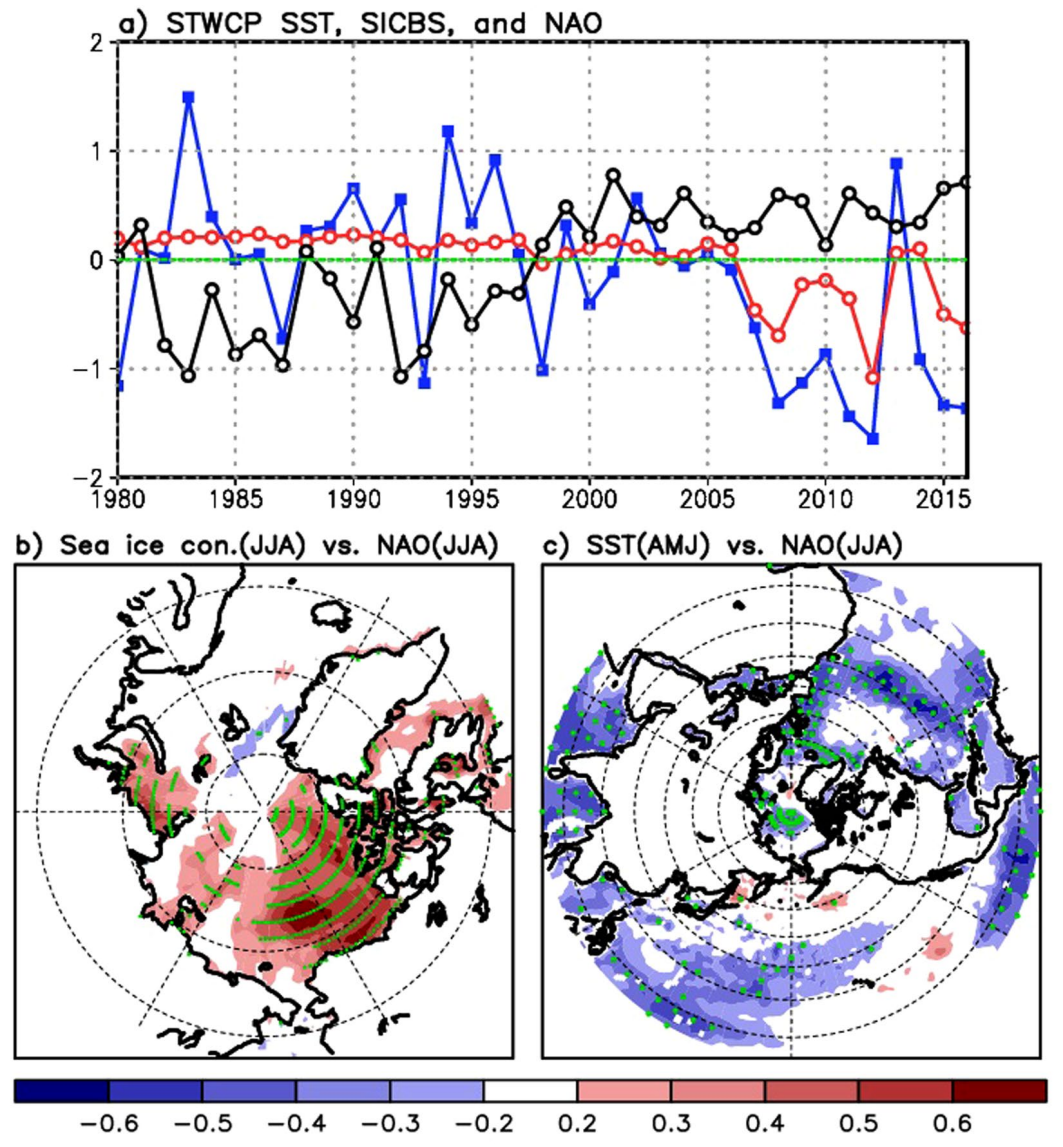
(**a**) Time series of the SST anomaly over the subtropical western-central Pacific (130°E–180°E, 0°–35°N) (black), the Arctic sea ice concentration (SIC) anomaly over the Beaufort Sea (180°–120°W, 75°–90°N) (red), and the phase of the NAO (blue) over the period 1980–2016. The NAO and SIC anomalies are averages for June, July, and August (JJA), while the SST anomaly is an average for April, May, and June (AMJ), which is two months before JJA to present time-lagged impact of the SST that leads the variation of SIC and NAO phase. Two spatial patterns represent (**b**) simultaneous correlations between the NAO indices and SIC for JJA, and (**c**) two month lag correlation between the northern hemispheric SST in AMJ and the NAO in JJA. Green dots are plotted, where correlations are statistically significant at 5 percent.

Boreal summer North Atlantic Oscillation (NAO) indices^1^ have tended to be in the negative phase in the last decade^2,3^ (Fig. 1a, blue line, in this paper), as characterized by high surface pressure anomalies over Greenland and low anomalies over the North Atlantic. The negative phase of the NAO has relevance to conditions in the Arctic and for the Greenland Ice Sheet in particular, as it enhances the potential for ice mass loss. Recent, severe surface melt in Greenland such as in 2007, 2010, and 2012^4–6^ occurred while the NAO was in its strong negative phase. An exceptionally strong positive phase of the NAO occurred in the summer of 2013, concurrent with a noticeable recovery of the Arctic sea ice cover^7^. The phase of the summer NAO has also been associated with Atlantic tropical cyclone activity on sub-seasonal time scales during the hurricane season^8,9^. The focus of this paper is to explore whether a remote impact of sea surface temperatures (SSTs) over the subtropical Pacific on the Arctic climate as characterized in earlier studies^10,11^ may explain the interannual variability of this index in summer, including the recent, frequent occurrence of the negative phase.

Previous studies have explored forcing mechanisms from both local and remote sources to account for Arctic-subpolar climate variability^12–14^. Arctic lower-tropospheric temperatures have been suggested to be largely a response to variations in Arctic sea ice cover^12,15^. Recent, enhanced Arctic warming has also been suggested to influence climate in the Northern Hemisphere mid-latitudes via changes in the jet stream and in the planetary-scale wave propagation^13,15–20^. But other studies have suggested that Arctic warming and the atmospheric response are largely explained as being forced remotely for example, by anomalous SST, and poleward heat and/or moisture fluxes in lower-latitude oceans^11,21–29^. This remote SST forcing was found to not only warm the Arctic mid-troposphere, but also trigger surface-based Arctic amplification through enhanced sea ice melt^23,30^.

These differing views on the relative roles of local and remote forcing mechanisms have also been applied to trends in the NAO index. Numerous studies have examined the potential for Arctic warming to force the negative phase of the NAO or its related Arctic Oscillation (AO) index^19,31–36^, while other studies have suggested the positive phase of the NAO in association with lower-latitude forcing^37–41^. The identification of the dominant forcing mechanism is consequential, as the expected amplification of high latitude conditions in a warming climate may suggest an associated change in the character of the NAO. Several modeling studies found little relation of the NAO phase in response to climate change^15,42^, while another study found a limited response that was confined to the summer season^43^. Potential changes in the NAO may become amplified by the effects of the associated circulation on the sea ice cover. Earlier studies reported that an upward trend in the NAO index was conducive to reduced Arctic sea ice cover during 1980s and 1990s^44–46^. But the exceptional Arctic sea ice cover reduction over the last two decades occurred despite the reversal in the NAO trend^47–50^, indicating a change in the physical relationship before and after ~2000.

In this study, we hypothesize that the physical linkage between lower-latitude forcing and the atmospheric response in the Arctic-subpolar region may characterize the atmospheric anomalies extending to the Arctic Atlantic sector and the vicinity of Greenland, contributing to a determination of the phase of the recent summer NAO^21^. For this study, we analyze reanalysis variables and observed sea ice concentration data, and perform atmospheric general circulation model (AGCM) experiments to 1) explore if the relationship between SST changes over the subtropical Pacific, variations in Arctic sea ice concentrations, and the phase of the seasonal mean NAO has become enhanced in recent summers and 2) address causality between the Pacific SST, the NAO, Arctic sea ice (i.e., time sequence of their occurrence), and their response time scales (time-lag). We also discuss the extent to which the variation of Arctic sea ice concentration and phase of the NAO in recent summers is correlated with the subtropical Pacific SST variation. We also examine the tendency towards a certain phase of the NAO under continued remote SST warming conditions by comparing observational and model results for recent summers and winters.

## Results

Time series in Fig. 1a evidence that the NAO (blue line) in summer (JJA) has been predominantly in the negative phase in the last decade: Since 2006, the seasonal mean NAO phase was negative during 10 of the 11 boreal summers. The Arctic SIC time series in Fig. 1a (red line) are shown for the Beaufort Sea region bounded by 180°–120°W and 75°–90°N (hereafter referred to as SICBS), as the SIC in that region exhibits a strong positive correlation with the phase of the summer NAO (see Fig. 1b). The SICBS exhibits a strong coherence with the NAO phase especially in 21st century summers (Fig. 1a), suggesting a possible connection between the negative (positive) phase of the NAO and sea ice reduction (increase)^51,52^.

**Figure 2:**
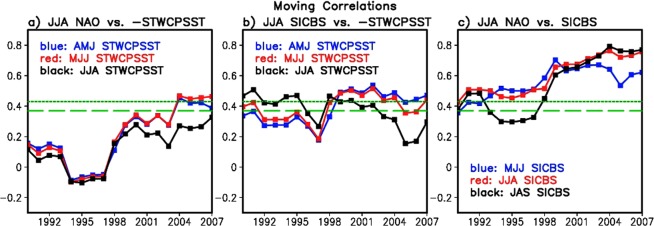
Interannual variation of lag–correlations (with 20 year running window) between the NAO, sea ice concentration over Beaufort Sea (SICBS), and the subtropical western–central Pacific SST (STWCPSST) over the period 1980–2016. Years on the x–axis denote the centers of the individual 20–year windows. Each panel shows time variation of the lag–correlations for (**a**) STWCPSST × (−1) in AMJ, MJJ, and JJA vs. JJA NAO, (**b**) STWCPSST × (−1) in AMJ, MJJ, and JJA vs. JJA SICBS, and (**c**) SICBS in MJJ, JJA, and JAS vs. JJA NAO. Trends are removed from the variables before calculating correlations. Short and long dashed lines in each panel represent a statistical significance limit at 5 percent (short-dash) and 10 percent (long-dash), respectively.

**Figure 3:**
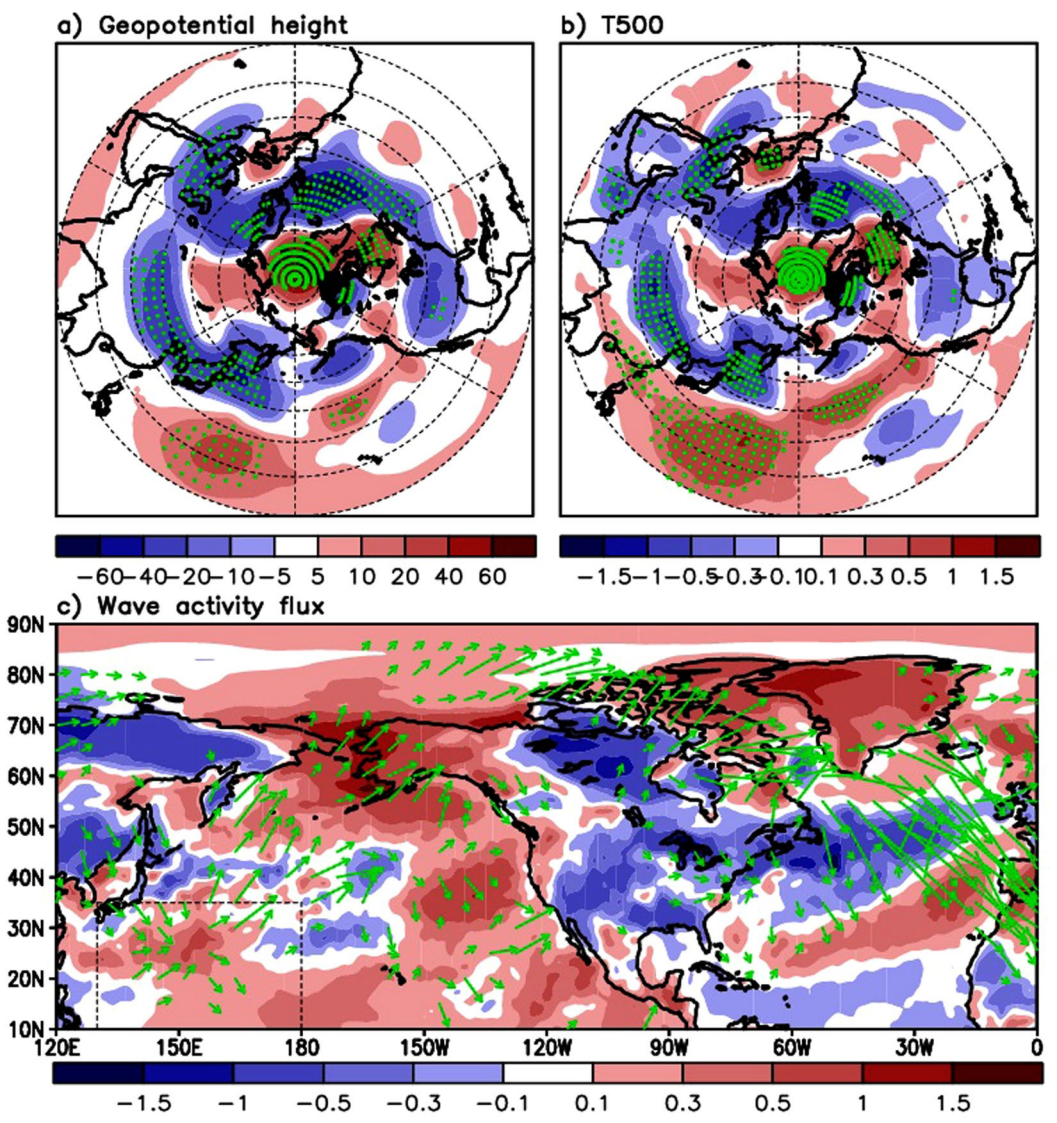
Upper panel: Differences in the recent boreal summer (the 21st century) upper–tropospheric geopotential height [m] (250hPa, left) and mid–tropospheric temperature [0.1 K] (500hPa, right) between the subtropical western–central Pacific SST (STWCPSST) positive and negative spring anomalies. Composite of geopotential height and temperature in summer (JJA) preceded by cooler than average STWCPSST (detrended) in spring is subtracted from the composite of the geopotential height and temperature in summer preceded by warmer than average STWCPSST in spring. Green dots are plotted, where the difference values are significant at 10 percent. Lower panel: Same as the upper panel but for wave activity flux vector distribution in summer at 250 hPa. Shaded is the difference in surface temperature (AMJ average) between the STWCP warming and cooling in spring. Box with dashed line denotes the STWCP region.

We find that the subtropical western-central Pacific SST (defined as 130°–180°E, 0°–35°N that encompass the subtropical region including the east/northeast of Philippine and part of the Western Pacific Warm Pool) (hereafter referred to as STWCPSST) two months earlier than JJA (black line in Fig. 1a) exhibits distinctively a negative relationship with the summer NAO in the 21st century (Fig. 1a), suggesting the possibility that the STWCPSST positive (negative) anomaly in spring could lead to a decrease (increase) in the NAO in summer. Figure 1c shows a distribution of lag-correlations between JJA NAO and Northern Hemisphere SSTs in AMJ (two months before JJA). Clearly, the STWCPSST is negatively correlated (correlations: the largest negative values of 0.4–0.5, statistically significant at 5 percent) with the NAO. Negative correlations are also found over the sub-tropical Atlantic, Indian Ocean, and Eastern Pacific, with the largest negative values similar to those for the STWCP. However, time variation of those lag-correlations demonstrates that correlations are the highest over the STWCP in recent decades. For example, the time-lagged correlation of the summer NAO as led by Indian Ocean SSTs and tropical Eastern Pacific SSTs, respectively, tends to increase with time, but does not clearly exceed the statistical confidence threshold (see Supplementary Fig. 1a,b, with blue denoted the AMJ period, and red denoting MJJ). For the Atlantic SST, we consider the cases when the summer NAO is led by the SST and lagged by the SST as their positive feedback was suggested in earlier studies^53^. These correlations find that the time-lagged correlation is slightly higher when the summer NAO leads the subtropical Atlantic SST than when the Atlantic SST leads the summer NAO (see Supplementary Fig. 1c). These features contrast with the correlations of the NAO with the STWCPSST as shown in Fig. 2a, in that they exhibit a higher correlation and a gradual increase with time with a 1–2 month lead (SST leads) than for the other oceans in recent decades, exceeding the statistical significance limit. Correlations are also higher when the STWCPSST leads the NAO, than simultaneous correlations (Fig. 2a), while the other oceans show lag-correlations (e.g., AMJ SST vs. JJA NAO) lower than simultaneous correlations (Supplementary Fig. 1). We also find that upper-tropospheric geopotential height and temperature anomalies in summer that are led by the positive SST values show a better organized negative NAO-like structure when the warm STWCPSST is considered, which we will discuss in more detail in Fig. 3 and Supplementary Figs 2 and 3. These characteristic features motivate experiments for imposing positive SST anomaly over the STWCP, as described in Methods Section.

Figure 2 shows time varying lag–correlations between the NAO and STWCPSST/SICBS (using a 20-year moving window), revealing that their relationships are particularly strong during the recent 21st Century summers. Note that the years on the x–axis denote the center of the individual 20–yr windows. Figure 2a demonstrates that the linkage between the STWCPSST positive (negative) anomalies and the negative (positive) phase of the summer NAO is clearer in the recent period, with the maximum correlation greater than 0.4 when the STWCPSST × (−1) leads the NAO by a month or two months (blue and red lines). Compared to them, simultaneous correlation is relatively low (black line). Figure 2b also supports the argument that the STWCPSST affects the variation of the SICBS in summer. Correlations close to 0.5 with statistical significance at 5 percent are found when the STWCPSST × (−1) leads the summer SICBS by one or two months (Fig. 2b). It may indicate that the STWCPSST variation in spring could explain about 25 percent (*r* ≈ −0.5) of the total variance of the summer SICBS.

**Figure 4:**
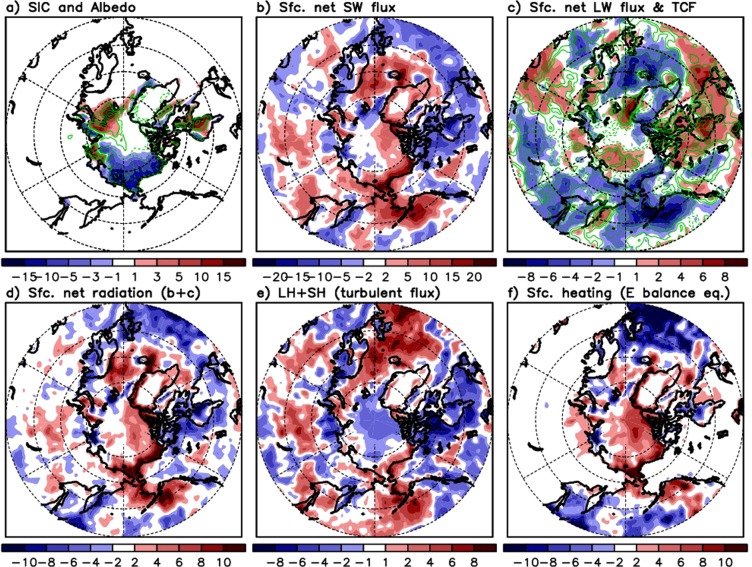
Same as Fig. 3 but for differences in (**a**) the Arctic sea ice concentration (shaded) and surface albedo (contoured) in percentage, (**b**) surface net shortwave downward flux [W m^−2^], (**c**) surface net longwave flux [W m^−2^] (shaded) and total cloud area fraction (contoured), (**d**) surface net radiation [W m^−2^], (**e**) turbulent fluxes (latent + sensible) [W m^−2^], and (**f**) surface net radiation minus turbulent fluxes ((**d**) minus (**e**)) to assess the energy balance [W m^−2^].

Figure 2c shows the moving correlations between the NAO and the SICBS. It is evident that the correlation gradually increases with time, reaching 0.8 in the 21st century. These high correlations are found at both positive and negative monthly lag, implying possibility of their strong linkage and positive feedback. For instance, the SICBS reduction and the resulting decrease in albedo (e.g., Fig. 4a) may allow for more absorption of solar insolation at the surface (e.g., Fig. 4b), driving warming and geopotential height increases in the Arctic atmosphere (mainly over the lower troposphere)^12,54^, which may be conducive to the negative phase of the NAO^51,52^. Alternatively, the geopotential height increase due to the remote impact of the STWCPSST increase leads to atmospheric warming in the Arctic via adiabatic subsidence^55^, resulting in the Arctic surface temperature increase and SIC reduction^10^. The relatively higher correlation shown with the black line (compared to the blue line) in the 21st Century in Fig. 2c indicates that the atmospheric process that leads the SICBS variation may be getting stronger than the opposite sequence that the SICBS leads the atmosphere. Overall, lag-correlation results in Fig. 2a–c are not inconsistent with our hypothesis that the STWCPSST in spring may play an increasing role in recent years to induce the large-scale atmospheric remote response in the Arctic^29^ in summer, modulating the SICBS and geopotential height anomalies in the Arctic including Greenland, linked to the phase of the NAO.

Figure 3 presents the large-scale atmospheric patterns in the Arctic for recent summers (e.g., the 21st Century) in response to the STWCPSST variation in spring. In Fig. 3a,b, we first separate the upper-tropospheric height and temperatures in JJAs into two groups based on the STWCPSST condition (i.e., positive or negative anomaly) in spring as a precursory signal. We then subtract the JJA height composite corresponding to the STWCPSST negative spring anomalies from the JJA height composite corresponding to the STWCPSST positive spring anomalies. The most pronounced feature in the difference map in Fig. 3a is a distribution of the height anomalies with alternate sign along the STWCP, northwestern Pacific and Aleutian Low region, and Arctic/Northeastern Canada, similar to the large-scale wave train.

The wave activity flux^56^ (WAF) pattern shown in Fig. 3c suggests the potential for a planetary-scale wave propagation from the STWCP where the local maximum of positive surface temperature anomalies are found (shaded in Fig. 3c) to the Arctic, and continuing into the North Atlantic. One may contend that WAFs provide a contribution from the western North Pacific, and this suggests a need to examine what occurs in that region in terms of wave generation. But vectors over the northeastern part of the STWCPSST may be understood to be the contribution of the SST, positive geopotential height (Fig. 3a), and temperature (Fig. 3b) anomalies over the STWCP. A large-scale distribution of SST, height, and temperature anomalies aligned with alternating sign from the STWCP up to the Arctic (through the mid-latitude western Pacific) together with the WAFs along that path indicates the feasibility that the wave activity starts near the STWCP. Linked to this wave propagation pattern, height increases over Greenland and lowering to its south are apparent (Fig. 3a), resembling the general pattern of the negative phase of the NAO. The corresponding temperature distribution in Fig. 3b is consistent with the height anomaly distribution, producing the negative temperature anomaly over the northwestern Pacific/Aleutian Low and North Atlantic region and strong positive anomaly in the Arctic, Greenland, and Northeastern Canada, as well as the STWCP region.

We also calculate differences in the summer upper-tropospheric height and temperatures for the Indian and Eastern Pacific tropical/subtropical ocean SSTs in spring. The results (Supplementary Figs 2 and 3) find positive height and temperature anomalies over the part of Arctic and/or Greenland region, along with the negative (Supplementary Fig. 3) or still the positive anomalies (Supplementary Fig. 2) over the south of Greenland. However, those anomalies less clearly indicate the pattern of the NAO negative phase. This contrasts with the pattern resulting from the STWCPSST forcing shown in Fig. 3. We additionally calculated for the subtropical North Atlantic SSTs in spring. The result in Supplementary Fig. 4 shows similarity to the negative phase of the NAO as the whole of Greenland is characterized by the positive height anomaly and warmer condition while the opposite is found over the southeast of Greenland. However, as it is addressed earlier, focus of this study is on the STWCP, where the role of SST leading the NAO phase is stronger than the opposite sequence, unlike the North Atlantic case.

We further investigate the Arctic response to the STWCPSST by considering the surface energy balance (e.g., radiative and turbulent fluxes). Composite anomalies are calculated for the radiative and turbulent fluxes, in the same way as done in Fig. 3. We first diagnose the JJA SIC distribution. It is evident from the difference map that the STWCPSST positive anomaly is followed by a reduction in SICBS in summer (Fig. 4a), when the height and temperature both increase in the Arctic as shown in Fig. 3. The SIC loss indicates a decrease in surface albedo (contour lines in Fig. 4a) and suggests the possibility of more absorption of the incoming shortwave flux. This is confirmed in Fig. 4b, which shows that the surface net shortwave flux over the Arctic increases specifically over the Beaufort Sea.

In contrast, the surface net downward longwave flux near the coast of Beaufort Sea does not increase after the STWCPSST increase. The net longwave flux is lower in that region when the STWCPSST is warmer than average in spring, while the Arctic open ocean region shows an increase (Fig. 4c). Contour lines in Fig. 4c show that this net longwave flux pattern is consistent with the total cloud area fraction. Overall, the net longwave flux tends to be lower (higher) and thus have cooling (warming) effects over less (more) cloudy regions, where a higher (lower) net shortwave flux is observed. The surface net radiation defined as the sum of surface net shortwave and longwave fluxes reveal the increased net radiation over the Arctic in response to the STWCPSST increase (Fig. 4d). Consistent with previous studies^57,58^ that explain the increase in the net solar flux into the Arctic in summer due to sea ice retreat by virtue of the ice-albedo feedback, it is apparent in Fig. 4b,c that the shortwave flux plays an important role in this increased surface net radiation. The pattern of turbulent fluxes including the latent and sensible heat fluxes show that these two fluxes are smaller over the Arctic and northern part of Greenland and larger over the mid-latitude Atlantic under the condition of STWCPSST increase. We see some positive difference value of turbulent fluxes along the coastal region of Beaufort Sea, but the magnitude is smaller than that seen in the surface net radiation, indicating that energy surplus is expected in that region, based on the energy balance equation. Subtraction of turbulent fluxes from the net radiation (i.e., Fig. 4d minus Fig. 4e) demonstrates that the Arctic and Greenland region has the surplus energy to heat surface (red shaded in Fig. 4f), while the mid-latitude Atlantic region does not. Positive/negative signs of this difference pattern across the Arctic and the Atlantic are very similar to the temperature/geopotential height anomaly distribution observed during the negative phase of the NAO (e.g., Fig. 3a,b).

**Figure 5:**
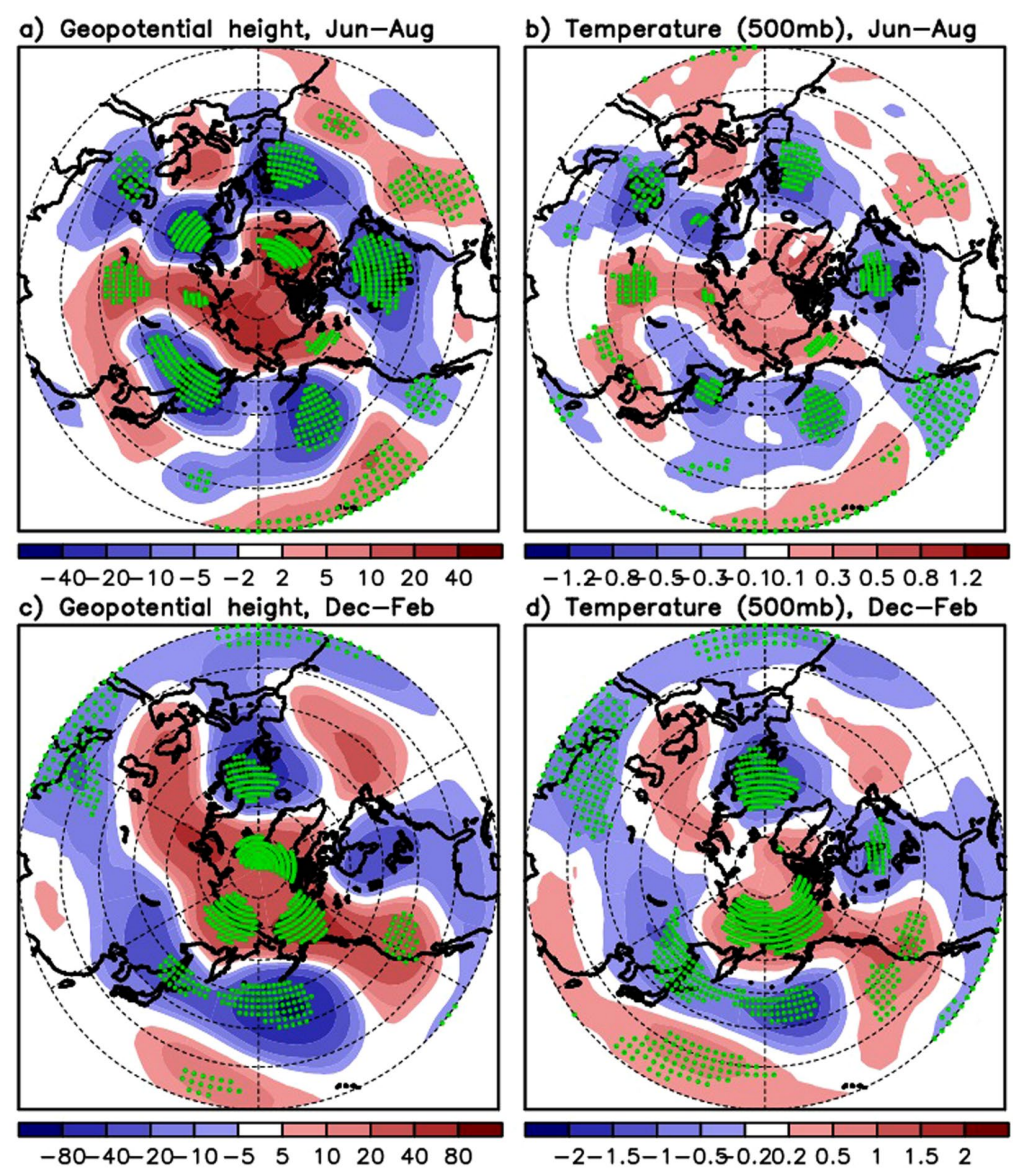
Responses of geopotential height and temperature to the subtropical western–central Pacific SST (STWCPSST) increase simulated by the atmospheric general circulation model. Upper panel: Difference in 20 member averaged Jun–August geopotential height and temperature between Exp. SPW, where the observed warming (SST average over the years when the SST is warmer than climatological average) is imposed over the STWCP in spring, and Exp. CTL forced by climatological SST everywhere. Lower panel is the same as upper panel but for the response in winter to the STWCPSST increase in fall. Green dots are plotted, where the difference values are significant at 10 percent.

We next conduct the AGCM experiments to clarify if tropospheric height and temperature increases in the Arctic may in fact be driven by the STWCPSST increase alone in the preceding season. Note that the result for winter is added here. As described in Methods section, the GEOS-5 AGCM is forced by the observed positive SST anomaly in spring and fall imposed over the STWCP region (Exp. SPW). The observed STWCPSST positive anomalies in spring and fall, respectively, are estimated by averaging SST over the years when the SST is greater than climatological average. Climatological SSTs and sea ice concentrations (climatology from average over 1980–2015) are prescribed elsewhere. This experiment is intended to clarify the impact of the STWCPSST positive spring (fall) anomalies on the Arctic-subpolar atmosphere in summer (winter). The left panel in Fig. 5 represents the averaged upper-level geopotential height anomalies from a 20-member ensemble for summer (Fig. 5a) and winter (Fig. 5c). Note that the anomaly field indicates differences with the geopotential heights simulated in the Exp. CTL, where a full climatological SST and sea ice is prescribed everywhere. Figure 5a shows that the geopotential height field in summer is characterized by a positive anomaly over the Arctic and Greenland with a negative anomaly over the central-eastern midlatitude North Atlantic, resembling the negative phase of the NAO. The distribution of temperature also indicates warming in the Arctic and Greenland and relative cooling over the south of Greenland (Fig. 5b). Over the mid-latitudes Pacific, the spatial structure is characterized by negative height anomalies, along with positive anomalies to the south. We presume that a negative NAO-like pattern with somewhat smaller magnitude in Fig. 5b, compared to the observation, is partly due to 1) the absence in the warm STWCPSST forcing in summer (forcing is given only in spring) and 2) no impact from the Arctic sea ice anomaly (the prescribed sea ice is climatology) on atmosphere. We also see the pattern somewhat similar to the North Pacific Oscillation (NPO) characterized by a meridional dipole in the geopotential height (and sea level pressure) across the North Pacific/Bering Sea/Aleutian islands. However, it is not yet sufficiently clear from the available evidence to argue that the pattern is physically meaningful as our calculation of the lagged and simultaneous correlations between the observed STWCPSST and the observed NPO index turns out to be generally low (the maximum of ~0.25 in the 21st century).

Similar responses were found in the Arctic for the winter season (Fig. 5c,d). This shows that, given the observed SST increase above climatology over the STWCP, the atmospheric response in the Arctic is an increase in geopotential height and temperature^10^, consistent with the observed distribution for the 20th century winter shown in Supplementary Fig. 5a,b. This height and temperature increase extends to Greenland, providing favorable conditions for constructing the negative phase of the NAO. Physically linked to this NAO phase, the negative height and temperature anomalies are found in the eastern Atlantic and western Europe^52,59^. Overall, the model experiment results presented here clarify that it is the lagged atmospheric response in the Arctic-subpolar region to the STWCPSST that contributes to development of the negative phase of the NAO.

## Summary and Discussion

Observational evidence and AGCM experiments shown in this study suggest a physical linkage between the STWCPSST springtime positive anomalies and the frequent occurrence of the negative phase of the NAO in recent summers. An increase in the tropospheric height and temperature fields with 0–2 month lag is more pronounced in 21st Century summers in the Arctic in response to the positive STWCPSST anomaly in spring. This atmospheric response extends to Greenland, providing favorable conditions for the negative phase of the summer NAO, which has been prominent in the 21st Century. A lagged large-scale atmospheric response to an STWCPSST positive (negative) anomaly is closely connected to Arctic SICBS reduction (recovery) and the radiative/turbulent energy fluxes that reinforce (weakens) Arctic warming. Based on time-varying correlations between the NAO and the SICBS for the 21st Century summer period, we suggest that the NAO-related atmospheric response to the STWCPSST leads the SICBS variation first, and then the SIC provides a positive feedback^60^ to the atmosphere via atmosphere/ice/ocean interaction processes.

This study focuses more on the STWCPSST than the SSTs in the other tropical/subtropical oceans. This does not indicate that the NAO phase is related to the STWCP region SST only. We acknowledge that, based on the results, the Arctic atmosphere and NAO may be also associated with the tropical/subtropical SST over the Indian, Eastern Pacific, and Atlantic, as well as the STWCP region. This analysis finds that the SST over the STWCP is more significantly related to the NAO during recent years as compared to the tropical SST of other locations. Also, time lagged-relationship between the spring SST and the summer NAO is higher than simultaneous relationship for the STWCPSST, while that feature is not clearly found for the other oceans.

An intriguing aspect of Fig. 2 and Supplementary Fig. 1 is the gradual increase with time of the moving correlations over the past four decades. This trend is coincident with greater interannual variability in the SICBS in recent years. During the 20th century, for example, the interannual variance of the SICBS was very small, possibly due to the thicker and more rigid ice distribution over the Arctic than in the 21st century (red line in Fig. 1a). As high latitude warming advanced in the 21st century, the Arctic sea ice cover became thinner and the ice cover became more mobile, perhaps resulting in an ice pack^61,62^ more responsive to the remote forcing of the STWCPSST. It is speculated that this response of the SICBS may have contributed to the gradual increase in the lagged correlations between the STWCPSST, SICBS, and the NAO clearly presented in Fig. 2. However, further work would be required to reach a firm conclusion.

The results of this study may raise a question regarding whether the phase of the NAO is more likely to be negative in a continued climate change condition that exhibits an upward SST trend over the subtropical Pacific. While the results for the 21st Century summer period and the AGCM experiments appear to suggest a tendency toward a more negative phase of the NAO in response to the STWCPSST increase, it is shown that the observed recent winters in the 21st Century do not experience the frequent negative phase of the NAO despite the upward SST trend in the Pacific. For example, an additional analysis for the observed winters finds that, while the negative phase of the NAO pattern was dominant in the late 20th century winters (1980–1999) following the STWCP increase in fall (Supplementary Fig. 5a,b), the pattern in the Arctic in the 21st Century (2000–2016) is quite different with a significance at 5 percent, sometimes leading to near zero or even the positive phase of the NAO (Supplementary Fig. 5c,d). Another example that did not clearly present the negative phase of the NAO recently is summer 2017. As the first exceptional case in the 21st Century summer, the positive geopotential height anomalies were not so dominant in the Arctic and Greenland^63^ in the presence of the strong positive STWCPSST anomaly in spring-summer 2017. As a result, the NAO was not clearly in the negative phase (it was in near-zero phase) in June/July, though the neighboring months of May and August in summer are clearly in a negative phase^1^. It has been generally accepted that the observation reflects both the response to the SST forcing and unforced natural variability (Supplementary Fig. 5c,d), while the multi-member mean AGCM result primarily presents a response to the SST forcing (Fig. 5c,d). Based on this difference, we speculate that the lack of a strong positive height anomaly in the Arctic and negative NAO in recent winter observations during the STWCPSST increase is partly due to the unforced natural variability (internal atmospheric noise), which is generally unpredictable by the model. However, further investigation should be needed to have more confirmative answer.

It has been understood that the phase of the NAO is determined by many factors, and is not driven by the STWCPSST anomaly alone. For example, some earlier studies suggested the partial relationship of the NAO phase with the Atlantic SST condition including the Atlantic Multidecadal Oscillation (AMO)^38^. It would be also possible that the atmospheric response to the STWCPSST is masked by the other NAO development mechanism arisen from internal atmospheric processes^64^, resulting in not necessarily the negative phase of the NAO in the presence of the STWCPSST increase. A series of results (e.g., recent summer and model experiments) and discussions in this study suggest that geopotential height increase and warming in the Arctic, SICBS reduction, and negative phase of the NAO is indeed possible in response to the STWCPSST increase. However, the Arctic atmospheric response and the NAO phase in reality is not determined only by the STWCPSST condition. Thus, the negative phase of the NAO would not be guaranteed in a continued STWCPSST warming condition in the future as long as the impact of the STWCPSST warming does not overwhelm the counteracting influences.

## Data and Methods

### Observation and reanalysis data

The observed Arctic sea ice concentration data at 1° longitude-latitude resolution for the period 1980 through present (2017) are obtained from HadISST.2 http://www.metoffice.gov.uk/hadobs/hadisst2/data/download.html ^65^. The observed SST prescribed in AGCM experiments consist of the NOAA Optimal Interpolation Sea Surface Temperature (OISST) data^66^.

The atmospheric reanalysis dataset used is NASA Modern-era Retrospective Analysis for Research and Applications, version 2 (MERRA–2)^67^ at 0.625° longitude × 0.5° latitude resolution. The primary variables used for this study consist of geopotential height, temperature, and wind at tropospheric pressure levels (850, 700, 500, and 250 mb)^68^, cloud fraction, surface albedo, short and long wave fluxes at surface^69^, and turbulent fluxes (e.g., latent and sensible heat fluxes) at surface^70^.

### Methods (Numerical model and experimental design)

NASA Goddard Earth Observing System model, version 5 (GEOS-5) is used for the model experiments. In order to explore large-scale atmospheric responses to idealized SST over the subtropical Pacific, we try uncoupled atmospheric GCM (AGCM). The model has 72 hybrid sigma-pressure vertical levels, extending to 0.01hPa, and 0.5° latitude/longitude horizontal grid spacing. The convection scheme is a modified version of the Relaxed Arakawa Schubert (RAS) scheme of^71^. It includes a stochastic Tokioka trigger function^72,73^ that governs the lower limits on the allowable entrainment plumes^73–75^. The model has the option for a standard single-moment microphysics^76^ or a two-moment cloud microphysics^77^ embedded within the RAS convective parameterization, and the simulations described here used the single moment option. Further details about the GEOS-5 AGCM can be found in^75,78^.

We carried out AGCM experiments to identify the impact of the subtropical Pacific warming on the global atmospheric circulation. We first conducted climatological SST–forced runs as a control experiment (Exp CTL). The SSTs are prescribed to be climatological (SST are averaged over the period 1980–2015) everywhere. The main experiment (Exp SPW) (here SPW stands for “subtropical Pacific warming”) is forced by the same SST as Exp CTL, except over the STWCP region (130°–180°E, 0°–35°N) where the observed warming (SST averages over the years when the SST is warmer than climatological average) has been added to climatology to include the warming effect. Specifically, this warming is imposed only for spring (MAM) and fall (SON) seasons to explore their warming impact on the atmospheric responses in the following seasons of summer (JJA) and winter (DJF). Model is initialized in February and integrated through February next year. The 20 ensemble members are distinguished by having different atmospheric/land initial conditions taken from MERRA–2 during the 20-day period February 9 through February 28.

## Supplementary information

Supplementary Information

## Data Availability

The MERRA-2 datasets are archived at the Goddard Space Flight Center Distributed Active Archive Center (GSFC DAAC). Geopotential height, temperature, and wind variables used in this study can be downloaded from 10.5067/2E096JV59PK7. Cloud and radiative flux variables are available at 10.5067/H3YGROBVBGFJ, and the surface turbulent flux variables are obtained from 10.5067/0JRLVL8YV2Y4. The observed Arctic sea ice concentration data are downloaded from HadISST.2 http://www.metoffice.gov.uk/hadobs/hadisst2/data/download.html. The observed SST prescribed in AGCM experiments consist of the NOAA Optimal Interpolation Sea Surface Temperature (OISST) data, available at https://www.esrl.noaa.gov/psd/data/gridded/data.noaa.oisst.v2.html.
