# Supplementary material for: Inter-relationship between subtropical Pacific sea surface temperature, Arctic sea ice concentration, and North Atlantic Oscillation in recent summers
Source: Sci Rep. 2019 Mar 5;9:3481. doi: 10.1038/s41598-019-39896-7 (PMC6401109; doi:10.1038/s41598-019-39896-7)
Supplement: Supplementary file 1 — Supplementary Information [file 41598_2019_39896_MOESM1_ESM.docx]

**Supplementary Information (Figures)**

**Inter-relationship between subtropical Pacific sea surface temperature, Arctic sea ice concentration, and North Atlantic Oscillation in recent summers**

**Young-Kwon Lim^1,4,*^, Richard I. Cullather^1,5^, Sophie M. J. Nowicki^2^, and Kyu-Myong Kim^3^**

^1^Global Modeling and Assimilation Office, NASA/GSFC, Greenbelt, Maryland

^2^Cryospheric Sciences Laboratory, NASA/GSFC, Greenbelt, Maryland

^3^Climate and Radiation Laboratory, NASA/GSFC, Greenbelt, Maryland

^4^Goddard Earth Sciences Technology and Research / I. M. Systems Group

^5^Earth System Science Interdisciplinary Center, University of Maryland, College Park, Maryland

*: corresponding author

Correspondence to [Young-Kwon.Lim@nasa.gov](mailto:Young-Kwon.Lim@nasa.gov) (Young-Kwon Lim)

Revised version submitted to Scientific Reports,

January 29, 2019

**
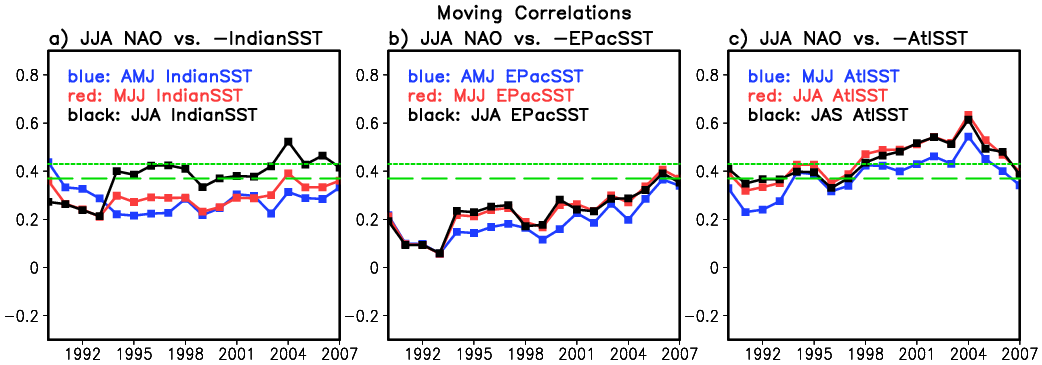
**

**Supplementary Figure 1**. Interannual variation of lag–correlations (with 20 year running window) between the NAO and the tropical Indian Ocean SST (60°–75°E, 5°–20°N) (a), the eastern Pacific SST (130°–100°W, 5°–15°N) (b), and the Atlantic SST (80°–50°W, 10°–25°N) (c) over the period 1980–2016. Years on the x–axis denote the centers of the individual 20–year windows. Each panel shows time variation of the lag–correlations for a) Indian Ocean SST×(–1) in AMJ, MJJ, and JJA vs. JJA NAO, b) Eastern Pacific SST×(–1) in AMJ, MJJ, and JJA vs. JJA NAO, and c) Atlantic SST×(–1) in MJJ, JJA, and JAS vs. JJA NAO. Trends are removed from the variables before calculating correlations. Short and long dashed lines in each panel represent a statistical significance limit at 5 percent (short-dash) and 10 percent (long-dash), respectively.

**
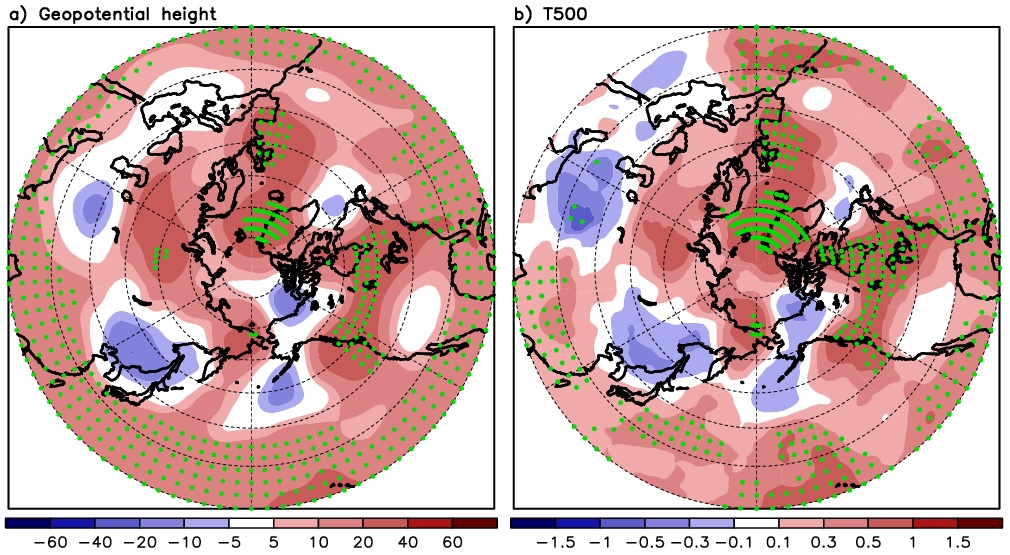
Supplementary Figure 2**. Differences in the recent boreal summer (~past 20 years) upper–tropospheric geopotential height [m] (250hPa, left) and mid–tropospheric temperature [0.1K] (500hPa, right) between the tropical Indian Ocean (60°E–75°E, 5°N–20°N) SST increase and decrease in spring. Composite of geopotential height and temperature in summer (JJA) preceded by cooler than average Indian Ocean (detrended) SST in spring is subtracted from the composite of the geopotential height and temperature in summer preceded by warmer than average Indian Ocean SST in spring.

**
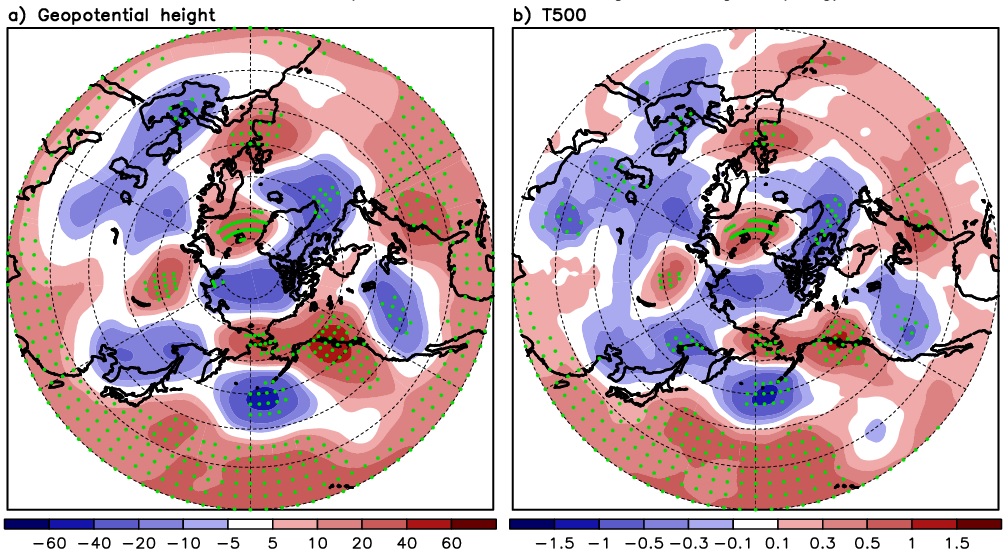
Supplementary Figure 3**. Same as the supplementary Figure 2 but for the sea surface temperature over the tropical Eastern Pacific (130°W–100°W, 5°N–15°N). Composite of geopotential height and temperature in summer (JJA) preceded by cooler than average Eastern Pacific (detrended) SST in spring is subtracted from the composite of the geopotential height and temperature in summer preceded by warmer than average Eastern Pacific SST in spring.

**
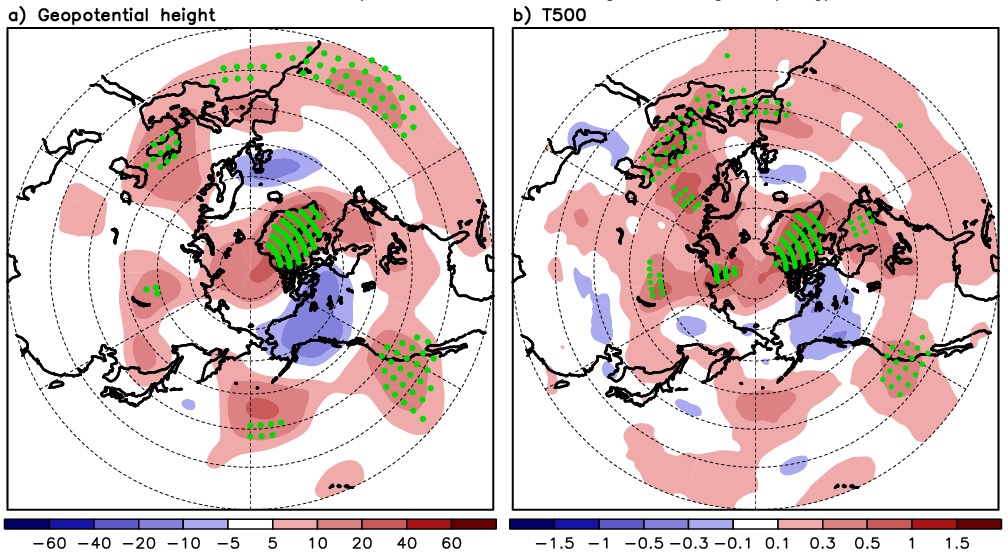
**

**Supplementary Figure 4**. Same as the supplementary Figure 2 but for the sea surface temperature over the subtropical North Atlantic (50°W–20°W, 20°N–30°N). Composite of geopotential height and temperature in summer (JJA) preceded by cooler than average subtropical North Atlantic (detrended) SST in spring is subtracted from the composite of the geopotential height and temperature in summer preceded by warmer than average subtropical North Atlantic SST in spring.


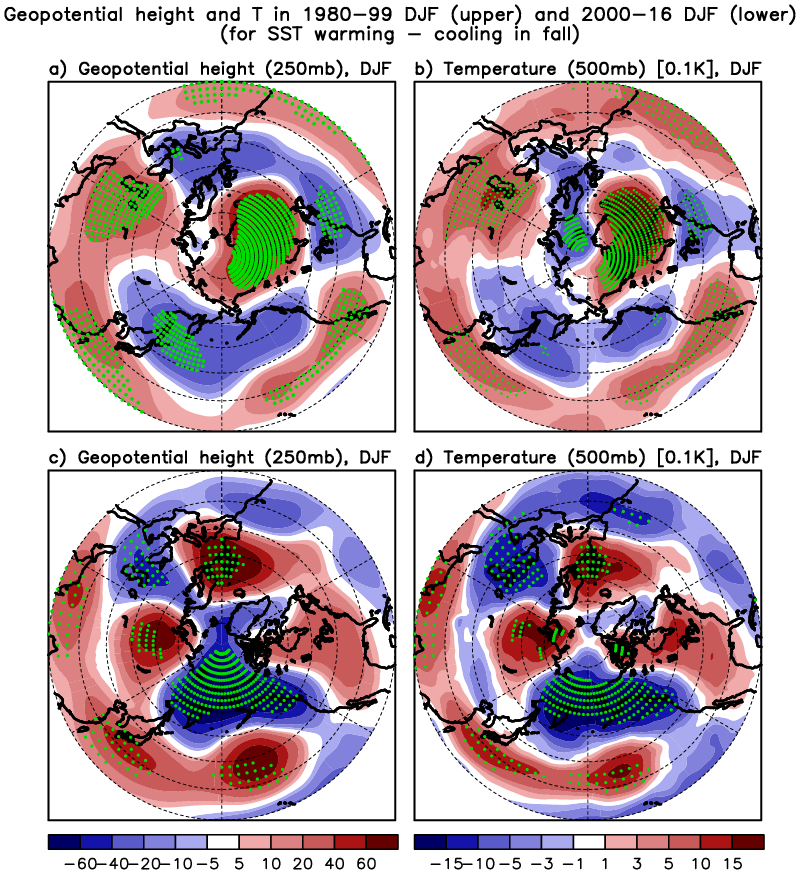


**Supplementary Figure 5**. Differences in the boreal winter upper–tropospheric geopotential height [m] (250hPa, left) and mid–tropospheric temperature [0.1K] (500hPa, right) between the subtropical western–central Pacific SST (STWCPSST) increase and decrease in fall. The upper panel represents the result for the early period 1980–1999, while the result for the recent period 2000–2016 is shown in the lower panel. Composite of geopotential height and temperature in winter (DJF) preceded by cooler than average STWCPSST (detrended) in fall is subtracted from the composite of the geopotential height and temperature in winter preceded by warmer than average STWCPSST in fall. Green dots are plotted, where the difference values are significant at 10 percent.
